# Higher Serum Testosterone Protects Against the Development of Type 2 Diabetes in Middle‐Aged but Not Older Men

**DOI:** 10.1111/dom.70885

**Published:** 2026-05-25

**Authors:** Mahesh M. Umapathysivam, Andrew D. Vincent, Jason Tan, Alebachew Ashagre, David Jesudason, Gary Wittert

**Affiliations:** ^1^ Freemason Centre for Men's Health and Wellbeing University of Adelaide Adelaide Australia; ^2^ Southern Adelaide Diabetes and Endocrine Service Adelaide Australia; ^3^ South Australian Health Medical Research Institute Adelaide Australia

**Keywords:** cohort study, observational study, population study, risk prediction, Type 2 diabetes

## Abstract

**Background:**

Low serum testosterone concentration has been reported to be causally linked to the risk of Type 2 diabetes (T2D). The benefit of T2D protection has been demonstrated to continue up to serum testosterone concentrations of 461 ng/dL (16 nmol/L). It is unclear if there is an upper threshold for this effect of testosterone nor whether this causal process is moderated by age, waist circumference (WC) or baseline glycaemia (A1c).

**Aim:**

To examine the effect of WC, age and baseline glycaemia on the relationship between testosterone and incident T2D risk.

**Methods:**

Men without T2D, cancer or testosterone treatment and with baseline and 5‐year follow‐up assessments in the Men Androgen Inflammation Lifestyle and Environment Study cohort were included. The association between baseline testosterone and 5‐year incident T2D was assessed using confounder‐adjusted logistic regressions, with and without restricted cubic splines and pairwise interactions with age, WC and baseline A1c. Primary analyses were in the complete case set, with multiple imputation being performed as a sensitivity analysis.

**Results:**

The analysis included 1299 men with 109 cases of 5‐year incident T2D (8.4%). An independent inverse relationship between serum testosterone concentration and 5‐year incident T2D was observed (OR = 0.92, 95% CI = [0.87, 0.97], *p* = 0.001). There were no detectable interactions between testosterone and either WC (*p* = 0.93) or baseline A1c (*p* = 0.38). There was, however, evidence for a strong interaction between testosterone and age (*p* = 0.003), with low testosterone being associated with elevated T2D risk only in younger men.

**Conclusions:**

These data suggest that higher concentrations of serum testosterone are protective against incident T2D in younger but not older men.

## 1 Introduction

Testosterone is the primary sex and anabolic hormone in men [1]. Some of the effects of testosterone, like the impact on muscle mass, are dose‐dependent, with the effect being independent of baseline serum testosterone concentration and with no established upper limit of effect [2]. In contrast, the effect of serum testosterone concentration on libido plateaus at a testosterone concentration of about 346 ng/dL (12 nmol/L) [3]. Whilst there is robust data demonstrating a strong association between pathologically low serum testosterone concentrations and incident Type 2 diabetes (T2D), the nature of the relationship between T2D risk and testosterone concentration within the normal range remains incompletely characterised.

Androgen deprivation therapy (ADT), which aims to reduce serum testosterone concentration below 50 ng/dL, provides a robust model of the impact of profoundly low testosterone on T2D risk. A meta‐analysis of studies comparing T2D risk suggested that ADT‐treated men, compared to non‐ADT‐treated men, had a 1.4‐fold increased risk of incident T2D [4]. Similarly, a meta‐analysis of cross‐sectional studies demonstrated that individuals with T2D have lower serum testosterone concentrations than individuals without T2D [5]. This is difficult to interpret given the bidirectional relationship between metabolic syndrome and testosterone concentrations [1]. Prospective cohort studies have been more informative but have largely dealt with testosterone in a binary fashion. For example, in the most definitive meta‐analysis to date by Ding et al., individuals were separated into the upper and lower halves of the testosterone range, and the incident T2D risk was compared [5]. Whilst the observed increase in T2D incidence is informative, it does not allow assessment of whether there is continued additive diabetes risk reduction with increasing testosterone exposure. A retrospective longitudinal study utilising available clinical measurements and serum testosterone concentration in a large primary care database is suggestive of a continuous inverse relationship between incident T2D and serum testosterone concentration but was unable to determine the contribution of potential confounders due to study design [6]. As such, it remains unclear if there is a continued reduction in T2D risk with higher testosterone concentrations across the normal range. Furthermore, the impact of traditional risk factors (e.g., glycaemia, adiposity and age) on a potential relationship between serum testosterone concentration and incident T2D is yet to be fully elucidated.

In this longitudinal study, the primary aim was to assess the nature of the relationship between baseline serum testosterone concentration and incident T2D risk at 5 years, including whether there was a continued beneficial effect of higher serum testosterone concentrations. The secondary aim was to determine if these associations were moderated by waist circumference (WC), baseline glycaemia and age.

## 2 Research Design and Methods

Participants for the analysis were selected from the Men Androgen Inflammation Lifestyle Environment and Stress (MAILES) study [7]. The MAILES study comprises suburban, community‐dwelling men aged 35–80 years at enrolment, randomly selected from the Northern and Western Local Areas of Adelaide, Australia. A more detailed description of the study design, procedures and recruitment was published previously [7]. In brief, 2563 age‐matched men from two existing prospective cohorts, the Florey Adelaide Male Ageing Study (FAMAS) [8] and the North West Adelaide Health Study (NWAHS) [9], were harmonised into a dataset incorporating detailed information on socio‐demographic, clinical, behavioural, chronic disease and medication data. The present study involved participants from MAILES 1 (2002–2006) and MAILES 2 (2007–2010). The FAMAS study was approved by the Human Research Ethics Committees of the Royal Adelaide Hospital (020305‐2002, 02035G‐2007) and the NWAHS study by the North‐Western Adelaide Health Service Research Ethics Committee (2004030‐2004, 2008034‐2008). Written informed consent was provided by all participants.

Incident diabetes was assessed 5 years after baseline assessment and was defined as glycated haemoglobin (HbA1c) ≥ 6.5%, a fasting glucose of ≥ 7.0 mmol/L, medication to lower blood glucose or a self‐reported clinical diagnosis of T2D.

### 2.1 Anthropometry

WC was measured to the nearest 0.1 cm using an inelastic tape maintained in a horizontal plane at the level of the narrowest part of the waist and read from the mid‐axillary line, with the subject standing comfortably with weight distributed evenly on both feet. WC was chosen as a marker of adiposity, as there is a closer relationship between WC and T2D risk and low serum testosterone than there is between BMI and these parameters [10]. Furthermore, with increasing age, the relationship between increasing BMI and T2D risk weakens, whereas it is retained with increasing WC [11].

### 2.2 Statistical Methods

We present means (SD) and frequencies (percentages) when presenting cohort demographics. A priori, we believed that serum testosterone concentration would affect diabetes risk and not vice versa [12, 13]. We follow the recommendations of VanderWeele for assessing causal inference by adjusting for all factors that may affect either the predictor of interest (serum total testosterone concentration) or the outcome (T2D) but not those that only affect the outcome via the predictor (i.e., instrumental variables) [14]. WC, age, lifestyle factors (smoking, alcohol and insufficient physical exercise) and low socio‐economic status are known to cause or are surrogates for causes of reduced testosterone and increased risk for T2D [15]. Family history of T2D and baseline A1C are known to increase the risk of T2D at 5 years [7]. These factors are thereby included in all assessments of the association between testosterone and incident T2D. Associations are assessed using multivariable binomial logistic regressions. The hypotheses of interest, whether age, baseline A1c or WC moderate the testosterone‐T2D association, were assessed by inclusion of pairwise interactions between total testosterone and each of these three factors. Figure S1 presents a directed acyclic graph depicting the believed causal processes and moderating hypotheses. The potential for non‐linearity in the associations of testosterone and continuous confounders with incident T2D was assessed by inclusion of restricted cubic splines with 4 degrees of freedom. Likelihood ratio tests of the linear (without interaction) model nested within extended models were used to assess interactions and non‐linearity. The primary analyses were within the complete case analysis set (CC), with sensitivity analyses being performed by repeating these analyses, imputing missing covariate data using multiple imputation (MI). We included all three pairwise interactions in the imputation model, thereby ensuring that the analysis model was contained within the imputation model. Significance in all analyses is set at a threshold of 0.05 (two‐sided) with no adjustment for multiple testing. Analyses were performed using R v4.4.2 with the packages *splines* and *mice* for analyses and *scales* for graphical presentation.

## 3 Results

### 3.1 Cohort Summary

A total of 2563 men in MAILES were available for inclusion, of which 1299 were included in the analysis set. Of the 1264 excluded, 737 did not have both baseline and 5‐year follow‐up assessments, 356 had a diagnosis of diabetes at baseline, 96 had a cancer diagnosis, 65 had an overt hypogonadism requiring treatment at baseline and 10 reported use of testosterone or oestrogen during the follow‐up period. In the analysis set, there were 109 cases of incident diabetes (8.4%). The mean (±SD) serum total testosterone concentration in the cohort was 17.37 ± 5.6 nmol/L, the mean age was 53.2 ± 10.9 years, the mean WC was 99.63 ± 11.4 cm and the mean HbA1c was 5.53% ± 0.37% (Table 1). The subgroup of men aged 61–79 years reported lower rates of family history of T2D and higher baseline glucose levels and exhibited greater rates of incident T2D while having similar levels of total testosterone as men aged 35–60 years (Table S1).

**TABLE 1:** Baseline demographic summary statistics and incident Type 2 diabetes.

	*N* = 1299	Missing (%)
Age		
Mean (SD)	53.17 (10.89)	
BMI (kg/m^2^)		
Mean (SD)	28.17 (4.23)	1 (< 1%)
Weight (kg)		
Mean (SD)	86.43 (14.39)	1 (< 1%)
WC (cm)		
Mean (SD)	99.63 (11.35)	2 (< 1%)
Smoking status		
No	1042 (80%)	5 (< 1%)
Yes	252 (19%)	
Alcohol (std. drinks/day)		
Mean (SD)	2.31 (2.34)	80 (6%)
Physical exercise		
Sedentary	317 (24%)	83 (6%)
Low‐mod exercise level	756 (58%)	
High exercise level	143 (11%)	
SIEFA RSED score		
Mean (SD)	957.71 (65.76)	
Total testosterone (nmol/L)		
≤ 11	157 (12%)	
> 11–15	319 (25%)	
> 15–21	508 (39%)	
> 21	315 (24%)	
SHBG (nmol/L)		
Mean (SD)	32.17 (12.82)	40 (3%)
T2D family history		
No	861 (66%)	1 (< 1%)
Yes	437 (34%)	
Baseline A1c (%)		
3.4–5.5	675 (52%)	3 (< 1%)
> 5.5–5.7	257 (20%)	
> 5.7–6.1	308 (24%)	
> 6.1–6.4	56 (4%)	
Incident T2D		
No incident T2D	1190 (92%)	
Incident T2D	109 (8%)	

Abbreviations: A1c, glycated haemoglobin; BMI, body mass index; RSED, relative socio‐economic disadvantage; SD, standard deviation; SHBG, sex hormone‐binding globulin; SIEFA, socio‐economic indexes for areas; std., standard; T2D, Type 2 diabetes; WC, waist circumference.

### 3.2 Testosterone Association With Incident Diabetes

**FIGURE 1:**
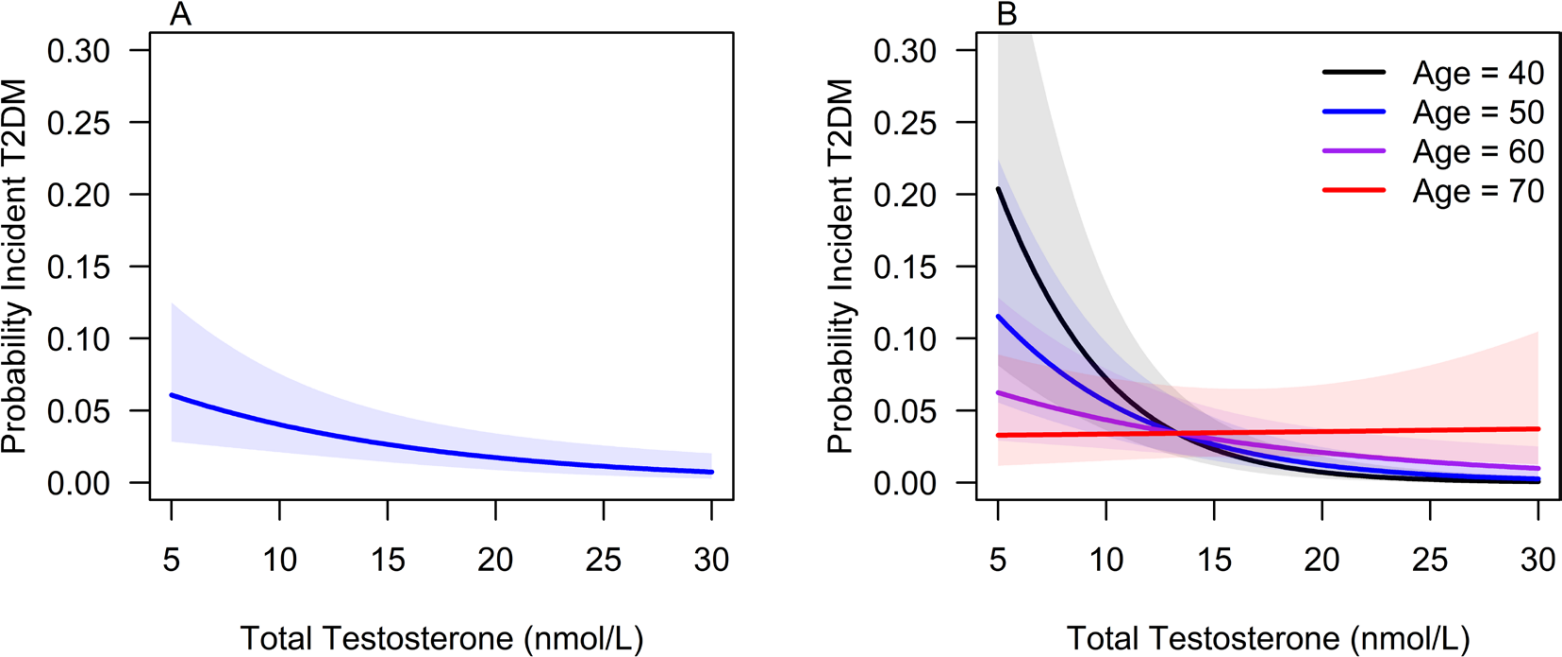
Estimated 5‐year incident T2D rates for increasing baseline serum testosterone concentrations (A) without and (B) with the pairwise interaction with age. The final model predicted incidence rates and 95% confidence bands for the complete case analysis presented in Table 2 for varying total testosterone and age. The estimates are for men aged 40, 50, 60 and 70 years who are non‐smokers with low‐moderate exercise levels and no family history of diabetes and other factors set at cohort mean levels.

Adjustment for confounding in a multivariable logistic regression indicated that the risk of incident T2D decreased with increasing total serum testosterone concentration (OR = 0.92, 95% CI = [0.87, 0.97], *p* = 0.001) in the complete case analysis set (Table 2 and Figure 1A). Including all individuals using MI to account for missing covariate information did not change this conclusion (Table S2). There was no evidence that the assumption of linearity on the logit scale was violated, nor was there evidence that the association between testosterone and T2D was influenced by possible non‐linearity of continuous confounders, including age (Table S3).

**TABLE 2:** Confounder‐complete‐case multivariable logistic regressions with and without the pairwise interaction between age and testosterone.

Predictor	Contrast	OR [95% CI]	*p*	OR [95% CI]	*p*
Intercept		0.04 [0.02, 0.08]	< 0.0001	0.03 [0.02, 0.07]	< 0.0001
Age		1.12 [0.87, 1.44]	0.38	1.40 [1.04, 1.87]	0.02
Waist circumference		1.02 [0.99, 1.04]	0.14	1.01 [0.99, 1.04]	0.22
Smoking status	Yes vs. no	0.59 [0.28, 1.23]	0.16	0.67 [0.32, 1.40]	0.28
Alcohol		0.87 [0.77, 0.98]	0.03	0.87 [0.77, 0.99]	0.03
Physical exercise	Low‐mod vs. sed	0.83 [0.48, 1.42]	0.49	0.82 [0.48, 1.42]	0.48
	High vs. sed	0.72 [0.29, 1.79]	0.49	0.76 [0.30, 1.90]	0.56
SES		0.09 [0.00, 3.26]	0.19	0.07 [0.00, 2.41]	0.14
Medication	Yes vs. no	1.25 [0.74, 2.10]	0.4	1.25 [0.74, 2.12]	0.4
Total testosterone (nmol/L)		0.92 [0.87, 0.97]	0.001	0.88 [0.82, 0.93]	< 0.0001
T2D family history	Yes vs. no	2.10 [1.28, 3.44]	0.004	2.12 [1.28, 3.51]	0.003
Baseline A1c		43.0 [18.2, 101.7]	< 0.0001	42.7 [17.9, 101.8]	< 0.0001
Age × total testosterone				1.08 [1.04, 1.13]	0.0003

Abbreviations: A1c, glycated haemoglobin; CI, confidence interval; Mod, moderate; OR, odds ratio; Sed, sedentary; SES, social economic status; T2D, Type 2 diabetes.

### 3.3 Moderating Effects

In both the complete case and multiply imputed analysis sets, there were no detectable pairwise interactions between testosterone and either WC (CC: *p* = 0.93; MI: *p* = 0.70) or baseline A1c (CC: *p* = 0.38; MI: *p* = 0.53). However, there was evidence for a strong pairwise interaction between total serum testosterone concentration and age (OR = 1.08; 95% CI = [1.04, 1.13]; *p* = 0.0003, Table 2). This interaction indicated that the elevated T2D risk with lower serum total testosterone concentration was apparent in younger men and greatly reduced in older men. Figure 1B presents the confounder‐adjusted estimates for the 5‐year predicted probability of incident T2D for men aged 40, 50, 60 and 70 years. Repeating the analyses with multiply imputed missing covariate data did not qualitatively change these conclusions (see Table S2).

## 4 Discussion

We assessed the relationship between baseline serum testosterone concentration and incident T2D risk at 5 years in a large cohort of community‐dwelling men with detailed characterisation of potential confounders. A robust inverse relationship between baseline serum testosterone concentration and T2D risk at 5 years in middle‐aged men, but not older men, was observed. In middle‐aged men, there appeared to be a continuous benefit from testosterone with decreasing risk of incident T2D over the normal range of serum testosterone concentrations. Whilst there was a strong interaction between baseline serum testosterone concentration and T2D risk with age, there was no detectable interaction between testosterone and either WC or HbA1c.

The observed benefit of testosterone is contrary to some previous studies, whereby the testosterone effect on T2D risk disappears with adjustment for adiposity factors [1, 16]. The data presented suggest that testosterone's effect on T2D risk is at least in part independent of baseline WC and HbA1c. The continued reduction in T2D risk with increased total serum testosterone concentration within the normal range suggests that increases in testosterone at both the lower end of the spectrum and the upper end of the spectrum will decrease the risk of T2D. This is an important observation as it suggests men may have reduced T2D risk with testosterone treatment, even if they have serum testosterone concentrations within the normal range. This is consistent with observations from the T4DM study, where individuals who, on average, had a low‐normal testosterone had a 40% relative reduction in T2D risk at 2 years if they were treated with testosterone compared to placebo [17]. This is a risk reduction comparable to that seen with metformin therapy in the Diabetes Prevention Program [18]. The reduction in incident diabetes with testosterone therapy in the T4DM study was reproduced in the Examining Outcomes in Chronic Disease in the 45 and Up Study [19]. The strong interaction between serum testosterone concentration and age on incident T2D risk suggests that the reduced risk of incident T2D with increased serum testosterone will be more pronounced in younger men.

Our study does not establish causality of the inverse association of testosterone and T2D risk; however, causality has been suggested by a prior interventional study (T4DM) [17]. Notably, the other large, randomised control trial, TRAVERSE, did not demonstrate a reduction in T2D risk [20]. This is the source of much controversy in the testosterone and diabetes field. Our study raises potential explanations for the discordant results of these studies. First, we observe a robust interaction between age and the relationship between diabetes and testosterone—the T4DM study men were younger (mean 59 years) than the men enrolled in the TRAVERSE study (mean 63 years), which did not detect an effect of testosterone on A1c. We also observed that the testosterone effect appeared linear on the logit scale, suggesting perhaps that the magnitude of a testosterone change, rather than baseline levels, determines the change in T2D risk. In the T4DM study, which used 1000 mg IM testosterone undecanoate, a mean trough testosterone concentration was 17.3 nmol/L (499 ng/dL), with an average testosterone level, therefore, well over 20 nmol/L (577 ng/dL), much greater than Traverse (13.45 nmol/L or 388 ng/dL). Based on our observations, a change in diabetes risk is likely independent of baseline T, and the recruitment of individuals with a higher mean testosterone in T4DM compared with Traverse (288 vs. 220 ng/dL) would likely not have had a large impact on the change in diabetes risk with testosterone treatment [20].

Previous studies in older men are not supportive of an association between serum testosterone and diabetes risk [21]; this is consistent with the data presented, where there was a strong interaction between age and testosterone and incident diabetes. The reason for this remains unclear. Potential reasons include a survivorship/ascertainment bias in that, as we excluded individuals with T2D at baseline, this may have preferentially selected individuals with low testosterone who had not developed T2D due to protective factors. Examination of this interaction in randomised control trials of testosterone therapy may minimise bias and allow greater confidence in this observation.

A potential limitation of the study is the age of the cohort (approximately 20 years), which may introduce uncertainty regarding extrapolation to the current population, which may have different characteristics; however, this limitation is offset by the observation of no significant difference between the study demographic and more recent cohorts [15].

There remain substantial gaps in the knowledge about the relationship between testosterone and risk of T2D, but existing data from RCTs may allow these to be addressed [22]. Specifically of interest is how age impacts the protective effects of therapeutic testosterone therapy on T2D risk [22]. The logical extension of the results presented is the retrospective analysis of randomised control trials of testosterone treatment that have demonstrated reduced T2D risk, to determine the importance of baseline testosterone and age on diabetes risk reduction associated with pharmacological testosterone treatment [23].

## 5 Conclusion

Higher baseline testosterone appears protective against incident diabetes independent of traditional risk factors at all levels of testosterone, but this effect diminishes as age increases. This is consistent with the recent observation that treatment with exogenous testosterone in men with low‐normal testosterone levels reduced the risk of incident T2D.

## Author Contributions

M.M.U. drafted the manuscript. A.D.V. extracted and analysed data and provided all statistical input. A.A. and J.T. reviewed the manuscript. D.J. jointly conceived the study idea and reviewed and assisted with manuscript production. G.W. jointly conceived the study idea and reviewed and assisted with manuscript production.

## Funding

This work was supported by the National Health and Medical Research Council (627227).

## Conflicts of Interest

Mahesh M. Umapathysivam received an unrelated honourarium from Boehringer Ingelheim for educational events. David Jesudason received an unrelated honourarium from Boehringer Ingelheim for educational events. Gary Wittert has received research funding from Bayer, Lilly and Lawley Pharmaceuticals; consulting fees from Besins, Sanofi and Wiley; speaker fees from Lilly, Amgen and Bayer; honourarium from Elsevier; and fees for expert reports from the Australian Health Practitioner Regulation Agency. The other authors declare no conflicts of interest.

## Supporting information


**Figure S1:** Directed acyclic graph of causal mechanisms and hypothesised moderating effects of waist circumference, age or baseline A1c. WC = Waist circumference; SES = Socio‐economic status; T2D = Type 2 diabetes; A1c = Glycated haemoglobin.
**Table S1:** Baseline demographic summary statistics and incident type 2 diabetes by age groups 35–60 vs. 61–79 years old. BMI = Body mass index; WC = Waist circumference; SIEFA = Socio‐economic indices for areas; RSED = Relative socio‐economic disadvantage; SHBG = Sex hormone‐binding globulin; A1c = Glycated haemoglobin; T2D = Type 2 diabetes; std. = standard; yr. = years; SD = Standard deviation.
**Table S2:** Confounder multiply imputed multivariable logistic regressions with and without the pairwise interaction between age and testosterone.
**Table S3:** The *p* values for likelihood ratio tests for the inclusion of non‐linear effects (restricted cubic splines with four degrees of freedom) for each continuous factor versus the linear implementation of these factors. Each factor was tested separately adjusting for the other predictors listed in Table 2, assessing the influence of non‐linearity of confounders with the linear total testosterone association with T2D risk.
**Table S4:** *p* values for the pairwise interactions of testosterone with age, waist circumference and baseline A1c in the complete case and multiply imputed data sets. The effect estimates of the age by testosterone interactions are presented in full in Tables 2 and S2.

**Data S1:** STROBE Statement—Checklist of items that should be included in reports of cohort studies.

## Data Availability

The data that support the findings of this study are available on request from the corresponding author. The data are not publicly available due to privacy or ethical restrictions.
