# Supplementary material for: Higher Serum Testosterone Protects Against the Development of Type 2 Diabetes in Middle‐Aged but Not Older Men
Source: Diabetes Obes Metab. 2026 May 25;28(8):6959–64. doi: 10.1111/dom.70885 (PMC13341335; doi:10.1111/dom.70885)
Supplement: Supplementary file 1 — Figure S1: Directed acyclic graph of causal mechanisms and hypothesised moderating effects of waist circumference, age or baseline A1c. WC = Waist circumference; SES = Socio‐economic status; T2D = Type 2 diabetes; A1c = Glycated haemoglobin. Table S1: Baseline demographic summary statistics and incident type 2 diabetes by age groups 35–60 vs. 61–79 years old. BMI = Body mass index; WC = Waist circumference; SIEFA = Socio‐economic indices for areas; RSED = Relative socio‐economic disadvantage; SHBG = Sex hormone‐binding globulin; A1c = Glycated haemoglobin; T2D = Type 2 diabetes; std. = standard; yr. = years; SD = Standard deviation. Table S2: Confounder multiply imputed multivariable logistic regressions with and without the pairwise interaction between age and testosterone. Table S3: The p values for likelihood ratio tests for the inclusion of non‐linear effects (restricted cubic splines with four degrees of freedom) for each continuous factor versus the linear implementation of these factors. Each factor was tested separately adjusting for the other predictors listed in Table 2, assessing the influence of non‐linearity of confounders with the linear total testosterone association with T2D risk. Table S4: p values for the pairwise interactions of testosterone with age, waist circumference and baseline A1c in the complete case and multiply imputed data sets. The effect estimates of the age by testosterone interactions are presented in full in Tables 2 and S2. [file DOM-28-6959-s001.docx]

**Supplementary Figure S1:** Directed acyclic graph of causal mechanisms and hypothesized moderating effects of waist circumference, age or baseline A1c. WC = Waist circumference; SES = Socio-economic status; T2D = Type 2 diabetes; A1c = Glycated hemoglobin


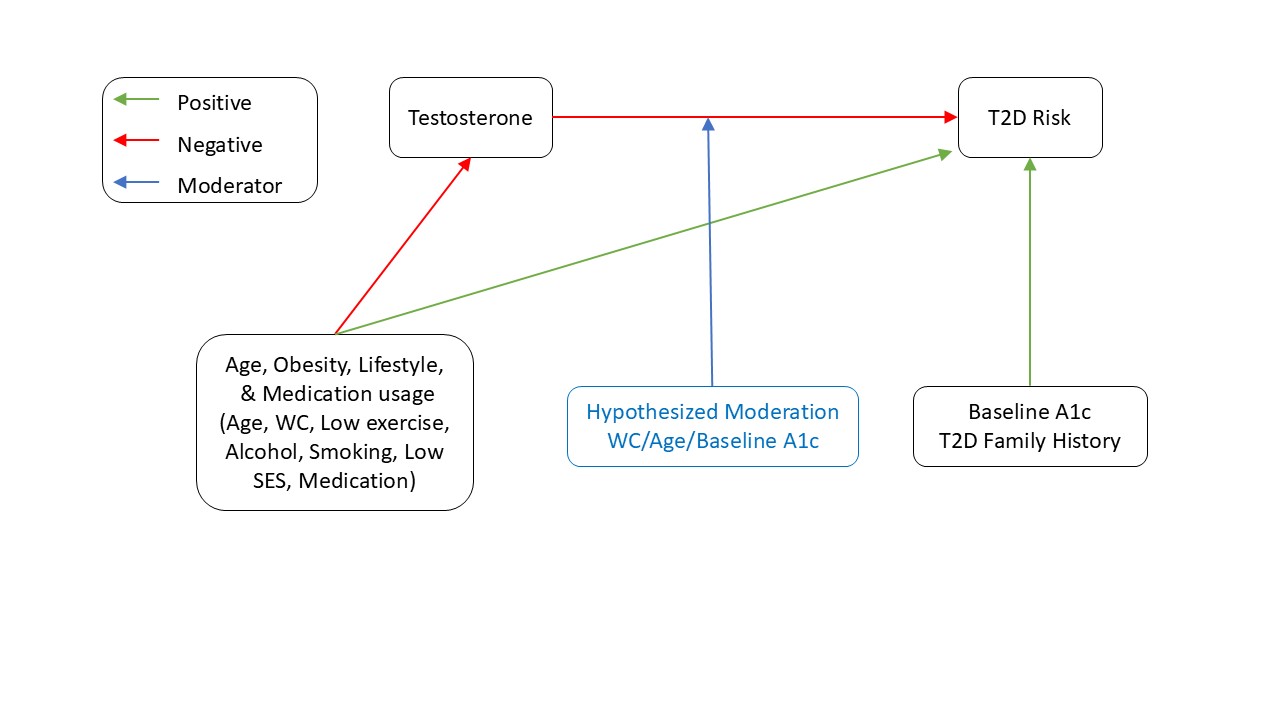


**Supplementary Table S1:** Baseline demographic summary statistics and incident type 2 diabetes by age groups 35-60 vs 61-79 years old. BMI = Body mass index; WC = Waist circumference; SIEFA = Socio-economic indexes for areas; RSED = Relative socio-economic disadvantage; SHBG = Sex hormone binding globulin; A1c = Glycated hemoglobin; T2D = Type 2 diabetes; std = standard; yr = years; SD = Standard deviation.

|  |  | **35-60yr** | **61-79yr** |
| --- | --- | --- | --- |
|  |  | **N = 964** | **N = 335** |
| **Age** |  |  |  |
|  | Mean (SD) | 48.08 (7.12) | 67.82 (4.99) |
| **BMI (kg/m2)** | |  |  |
|  | Mean (SD) | 28.3 (4.38) | 27.8 (3.74) |
|  | Missing | 1 ( <1%) | 0 ( 0%) |
| **Weight (kg)** | |  |  |
|  | Mean (SD) | 87.83 (14.8) | 82.43 (12.32) |
|  | Missing | 1 ( <1%) | 0 ( 0%) |
| **WC (cm)** | |  |  |
|  | Mean (SD) | 99.34 (11.76) | 100.48 (10.06) |
|  | Missing | 2 ( <1%) | 0 ( 0%) |
| **Smoking Status** | |  |  |
|  | No | 741 ( 77%) | 301 ( 90%) |
|  | Yes | 219 ( 23%) | 33 ( 10%) |
|  | Missing | 4 ( <1%) | 1 ( <1%) |
| **Alcohol (std. Drinks/day)** | |  |  |
|  | Mean (SD) | 2.4 (2.4) | 2.01 (2.13) |
|  | Missing | 50 ( 5%) | 30 ( 9%) |
| **Physical Exercise** | |  |  |
|  | Sedentary | 245 ( 25%) | 72 ( 21%) |
|  | Low-Mod Exercise Level | 550 ( 57%) | 206 ( 61%) |
|  | High Exercise Level | 112 ( 12%) | 31 ( 9%) |
|  | Missing | 57 ( 6%) | 26 ( 8%) |
| **SIEFA RSED Scores** | |  |  |
|  | Mean (SD) | 957.98 (65.15) | 956.93 (67.6) |
| **Total Testosterone (nmol/L)** | |  |  |
|  | <=11 | 116 ( 12%) | 41 ( 12%) |
|  | >11-15 | 232 ( 24%) | 87 ( 26%) |
|  | >15-21 | 391 ( 41%) | 117 ( 35%) |
|  | >21 | 225 ( 23%) | 90 ( 27%) |
| **SHBG (nmol/L)** | |  |  |
|  | Mean (SD) | 29.71 (11.53) | 39.17 (13.71) |
|  | Missing | 33 ( 3%) | 7 ( 2%) |
| **T2D Family History** | |  |  |
|  | No | 605 ( 63%) | 256 ( 76%) |
|  | Yes | 359 ( 37%) | 78 ( 23%) |
|  | Missing | 0 ( 0%) | 1 ( <1%) |
| **Baseline A1c (%)** | |  |  |
|  | 3.4-5.5 | 557 ( 58%) | 118 ( 35%) |
|  | >5.5-5.7 | 188 ( 20%) | 69 ( 21%) |
|  | >5.7-6.1 | 184 ( 19%) | 124 ( 37%) |
|  | >6.1-6.4 | 33 ( 3%) | 23 ( 7%) |
|  | Missing | 2 ( <1%) | 1 ( <1%) |
| **Incident T2D** | |  |  |
|  | No Incident T2D | 899 ( 93%) | 291 ( 87%) |
|  | Incident T2D | 65 ( 7%) | 44 ( 13%) |

**Supplementary Table S2:** Confounder multiply imputed multivariable logistic regressions with and without the pairwise interaction between age and testosterone.

| **Predictor** | **Contrast** | **OR [95% CI]** | **p-value** | **OR [95% CI]** | **p-value** |
| --- | --- | --- | --- | --- | --- |
| Intercept |  | 0.04 [0.02, 0.08] | <0.0001 | 0.04 [0.02, 0.07] | <0.0001 |
| Age |  | 1.09 [0.87, 1.38] | 0.46 | 1.28 [0.99, 1.67] | 0.06 |
| Waist Circumference |  | 1.02 [1.00, 1.04] | 0.12 | 1.01 [0.99, 1.04] | 0.18 |
| Smoking status | Yes v No | 0.62 [0.32, 1.21] | 0.16 | 0.66 [0.34, 1.30] | 0.23 |
| Alcohol |  | 0.88 [0.78, 0.99] | 0.03 | 0.88 [0.78, 0.99] | 0.03 |
| Physical exercise | Low-Mod v Sed | 0.85 [0.51, 1.43] | 0.55 | 0.85 [0.51, 1.44] | 0.55 |
|  | High v Sed | 0.67 [0.28, 1.64] | 0.39 | 0.70 [0.28, 1.71] | 0.43 |
| SES |  | 0.08 [0.00, 2.05] | 0.13 | 0.05 [0.00, 1.47] | 0.08 |
| Medication | Yes v No | 1.25 [0.77, 2.03] | 0.36 | 1.24 [0.76, 2.02] | 0.39 |
| Total Testosterone (nmol/L) |  | 0.92 [0.87, 0.96] | 0.0005 | 0.89 [0.84, 0.94] | <0.0001 |
| T2D family history | Yes v No | 1.86 [1.17, 2.96] | 0.009 | 1.87 [1.17, 2.99] | 0.009 |
| Baseline A1c |  | 46.8 [21.0, 104.3] | <0.0001 | 46.0 [20.6, 102.7] | <0.0001 |
| Age x Testosterone |  |  |  | 1.07 [1.02, 1.11] | 0.002 |
|  | | | | | |
|  | | | | | |
|  | | | | | |

T2D = type 2 diabetes, SHBG = sex hormone binding globulin, A1c = glycated hemoglobin; SES = Social economic status; OR = odds ratio; CI = confidence interval; Mod = Moderate; Sed = Sedentary.

**Supplementary Table S3:** The p-values for likelihood ratio tests for the inclusion of non-linear effects (restricted cubic splines with four degrees of freedom) for each continuous factor verses linear implementation of these factors. Each factor was tested separately adjusting for the other predictors listed in Table 2, assessing the influence of non-linearity of confounders with the linear total testosterone association with T2D risk.

|  | **NL p-value (df=3)** | **TT: OR [95%CI]** |
| --- | --- | --- |
| Total Testosterone (nmol/L) | 0.28 |  |
| Age | 0.96 | 0.917 [0.870, 0.965] |
| Waist Circumference | 0.59 | 0.918 [0.872, 0.967] |
| SES | 0.22 | 0.916 [0.870, 0.964] |
| Baseline A1c | 0.99 | 0.917 [0.871, 0.966] |

NL = non-linear; df = degrees of freedom; TT = total testosterone; OR = odds ratio; CI = confidence interval; A1c = glycated hemoglobin; SES = Social economic status

**Supplementary Table S4:** P-values for the pairwise interactions of testosterone with age, waist circumference and baseline A1c in the complete case and multiply imputed data sets. The effect estimates of the age by testosterone interactions are presented in full in tables 2 and S2.

|  | **CC: p-value** | **MI: p-value** |
| --- | --- | --- |
| Age | 0.0003 | 0.002 |
| Waist Circumference | 0.93 | 0.70 |
| Baseline A1c | 0.38 | 0.53 |

CC = complete case; MI = multiply imputed; A1c = glycated hemoglobin.
